# Serum Lactate Dehydrogenase as a Biomarker of Disease Burden and Chemotherapy Response in Canine High-Grade Multicentric Lymphoma

**DOI:** 10.3390/vetsci13010093

**Published:** 2026-01-17

**Authors:** Rafael Costa Bitencourt, Marina Franc Garcia, Adilson Paulo Marchioni Cabral, Tatiana Geraissate Gorenstein, Jéssika Cristina Chagas Lesbon, Letícia Abrahão Anai, Heidge Fukumasu, Rodrigo dos Santos Horta, Andrigo Barboza de Nardi, Aureo Evangelista Santana

**Affiliations:** 1 Department of Veterinary Clinic and Surgery, School of Agricultural and Veterinary Sciences, São Paulo State University (UNESP), Via de Acesso Professor Paulo Donato Castellane S/N—Vila Industrial, Jaboticabal 14884-900, SP, Brazil; marina.franc@unesp.br (M.F.G.); adilson.pm.cabral@unesp.br (A.P.M.C.); leticia.anai@unesp.br (L.A.A.); andrigo.barboza@unesp.br (A.B.d.N.); 2 Department of Veterinary Medicine and Surgery, Veterinary School, Universidade Federal de Minas Gerais (UFMG), Presidente Carlos Luz Avenue, 5162—Pampulha, Belo Horizonte 31310-250, MG, Brazil; taty_gorenstein@hotmail.com (T.G.G.); rodrigohvet@gmail.com (R.d.S.H.); 3 School of Animal Science and Food Engineering, University of São Paulo (USP), Duque de Caxias Norte Street, 225, Pirassununga 13635-900, SP, Brazil; jessika.chagas@usp.br (J.C.C.L.); fukumasu@usp.br (H.F.)

**Keywords:** B-cell lymphoma, biomarker, chemotherapy response, CHOP protocol, thrombocytopenia

## Abstract

Serum lactate dehydrogenase (LDH) is a recognized prognostic biomarker in human lymphomas, yet its clinical significance in canine lymphoma remains insufficiently characterized. This study aimed to quantify serum LDH levels in healthy dogs and dogs with high-grade multicentric lymphoma (ML) (predominantly B-cell) and to investigate correlations between LDH levels and established clinical and laboratory prognostic indicators. Twenty-seven dogs were prospectively enrolled: healthy controls (G1, n = 7) and dogs with high-grade ML (G2, n = 20). Immunophenotyping was performed by immunohistochemistry (CD3/CD79a). LDH concentrations were measured at diagnosis (T0) and after six weeks of CHOP-based induction chemotherapy (T1). Statistical analyses included Kruskal–Wallis, Wilcoxon signed-rank, Pearson’s correlation, and mixed-effects models. Dogs with high-grade ML exhibited significantly elevated LDH levels compared to controls (median 545.5 U/L, range: 288.2–2816 U/L vs. 143 U/L, range: 66–272; *p* < 0.001). Dogs with thrombocytopenia had higher baseline LDH (median 746 U/L, range: 612–921; *p* = 0.006) and greater reductions following chemotherapy (median −1011.7 U/L, range: −159 to −2064; *p* = 0.004). LDH levels declined significantly after treatment (overall median reduction 50.7%; post-chemotherapy range: 60.4–752 U/L; n = 15; *p* = 0.013), with normalization achieved in 77.8% of dogs with complete response versus 16.7% with partial or progressive disease (*p* = 0.02). We confirmed that serum LDH is significantly elevated in dogs with high-grade ML and declines following effective chemotherapy, supporting its utility as a dynamic biomarker of tumor burden and treatment response. Thrombocytopenic dogs may represent a biologically distinct subset warranting further investigation.

## Simple Summary

Lymphoma is a common type of cancer in dogs. Blood tests that track the disease during treatment are helpful for veterinarians. One such test measures an enzyme called lactate dehydrogenase (LDH), which can be higher in cancer cases, but its significance in dogs with lymphoma is not fully explored. This study found that dogs with aggressive lymphoma had much higher LDH levels compared to healthy dogs. After chemotherapy, LDH levels dropped, especially in dogs that responded well to treatment. Dogs that went into complete remission (CR) often had their LDH levels return to normal. Additionally, dogs with lower platelet counts had higher LDH levels at diagnosis and larger decreases during treatment. Overall, LDH is a simple, inexpensive blood test that can help monitor disease burden and treatment response in dogs with high-grade lymphoma.

## 1. Introduction

Lactate dehydrogenase (LDH) is an ubiquitous cytosolic enzyme catalyzing the reversible conversion of pyruvate to lactate, which plays a critical role in anaerobic glycolysis. In neoplastic cells, LDH is integral to the Warburg effect, wherein tumor cells preferentially utilize aerobic glycolysis even under normoxic conditions, promoting rapid proliferation, survival in hypoxic environments, and resistance to apoptosis [1,2,3,4]

Serum LDH activity reflects tissue turnover and cellular damage, with elevations documented in a range of malignancies, including lymphomas, leukemias, and germ cell tumors [5,6,7,8]. In veterinary oncology, LDH has been proposed as a potential marker for malignancy; however, the clinical utility and optimal application of LDH in canine lymphoma remains incompletely characterized. Previous studies have shown variable results, which may reflect differences in sample size, lymphoma subtypes, treatment protocols, and timing of LDH measurements [9,10,11].

Lymphoma is among the most prevalent hematopoietic tumors in dogs, representing up to 24% of all canine neoplasms and 83% of hematologic cancers [12]. High-grade multicentric lymphoma (ML) is the most common form, typically responding well to CHOP-based chemotherapy protocols. However, prognosis remains heterogeneous and depends on clinical stage, substage, immunophenotype, and biochemical abnormalities such as anemia, hypercalcemia, and potentially, serum LDH levels [12,13,14,15].

Despite evidence suggesting a prognostic role for LDH in canine lymphoma, its clinical utility remains underexplored, particularly in the context of dynamic changes during chemotherapy.

This prospective study aimed to: (1) quantify serum LDH concentrations in healthy dogs and dogs with high-grade ML; (2) investigate associations between LDH levels and prognostic factors; and (3) evaluate LDH dynamics during CHOP-based chemotherapy. We hypothesized that LDH would be significantly elevated in dogs with lymphoma compared to controls, would correlate with disease severity markers, and would decline following effective treatment, thereby serving as a clinically useful biomarker of disease burden and therapeutic response.

## 2. Materials and Methods

### 2.1. Ethical Approval

This study was conducted following approval from the Ethics Committee on Animal Use of São Paulo State University (protocol number 327/2024). Written informed consent was obtained from all pet owners prior to inclusion in the study.

### 2.2. Study Population and Patient Selection

Between March 2023 and October 2024, 34 dogs presenting with multicentric lymphadenopathy were screened for eligibility. Of these, 20 met all inclusion criteria and were enrolled in G2 (dogs with high grade ML).

The control group (G1) comprised 7 healthy mixed-breed dogs with a median age of 6.0 years (range: 2–11) and median body weight of 20.0 kg (range: 8.5–38.0). The group included 3 males (42.9%) and 4 females (57.1%), with 6/7 (85.7%) neutered. All dogs had ideal body condition scores (BCS 5/9) and normal hematologic and biochemical profiles.

For G2, inclusion criteria were: (1) cytological and histopathological diagnosis of high-grade ML confirmed by board-certified pathologist; (2) immunophenotyping by immunohistochemistry (CD79a/CD3); (3) clonal lymphoid population confirmed by polymerase chain reaction for antigen receptor rearrangement (PARR); (4) no prior chemotherapy or corticosteroid treatment (>1 mg/kg/day for more than 7 days) within 30 days; (5) measurable peripheral lymphadenopathy (≥10 mm); and (6) absence of concurrent neoplasms or severe systemic diseases that would preclude chemotherapy.

Reasons for exclusion (n = 14) included corticosteroid pretreatment at doses higher than 1 mg/kg/day for more than 7 days (n = 6); low-grade lymphoma (small cell, marginal zone) (n = 3); concurrent severe comorbidities precluding chemotherapy (n = 2); or owner declined treatment (n = 1).

### 2.3. Clinical and Laboratory Evaluations

At baseline (T0), all dogs underwent thorough clinical evaluation. All complete blood counts were performed using an automated hematology analyzer, and platelet counts were verified by manual blood smear examination performed by experienced clinical pathologists to exclude platelet clumping or analytical artifacts.

Serum biochemistry (alanine aminotransferase [ALT], alkaline phosphatase [ALP], total proteins, albumin, globulins, blood urea nitrogen [BUN], creatinine, ionized calcium, and total, direct, and indirect bilirubin) was analyzed using an automated biochemical analyzer (Labmax Plenno III^®^, Labtest Diagnóstica S.A., Lagoa Santa, MG, Brazil). Cardiac evaluation included echocardiography (MyLab™ 30Vet Gold, Esaote, Genova, Italy) and 6-lead electrocardiography (TEB^®^, São Paulo, Brazil) to exclude underlying cardiopathies that could confound LDH interpretation.

Serum samples were obtained after a 10–12 h fasting period and analyzed within two hours. All samples were visually inspected prior to analysis; none exhibited hemolysis or lipemia.

Dogs in G2 presenting thrombocytopenia underwent additional screening for vector-borne diseases *(Babesia* spp., *Ehrlichia* spp., and *Anaplasma* spp.) via serological and molecular diagnostics, all of which yielded negative results. For G2, a second LDH measurement was performed after six weeks of chemotherapy (T1) to assess dynamic changes associated with treatment response.

### 2.4. Serum LDH Measurement

Serum LDH activity was quantified using the UV-pyruvate-lactate method (LABORLAB^®^, São Paulo, Brazil), with the established reference interval for healthy dogs being 10–280 U/L [10].

LDH elevations were classified as mild (1–2× above the upper reference limit), moderate (2–3×), or severe (higher than 3×).

### 2.5. Definitions of Clinical Conditions

Anemia was categorized according to packed cell volume (PCV): mild (30–37%), moderate (20–29%), severe (13–19%), and very severe (<13%).

Thrombocytopenia was defined as platelet counts <175,000/μL, and thrombocytosis as >500,000/μL.

Body condition score (BCS) was assessed on a 9-point scale, with underweight defined as BCS < 4, ideal weight as BCS 4–5, and overweight as BCS > 5.

### 2.6. Clinical Staging and Substaging

G2 dogs were staged according to the World Health Organization (WHO) classification system for canine lymphoma, ranging from stage I (single lymph node involvement) to stage V (bone marrow or extranodal organ infiltration). Substaging (A or B) was based on the presence or absence of clinical signs [16]. Thoracic radiographs (3 incidences) and abdominal ultrasonography were performed for metastatic assessment. Bone marrow aspiration was conducted based on the presence of cytopenias or circulating atypical lymphocytes.

Immunophenotyping was performed on formalin-fixed, paraffin-embedded lymph node sections using anti-CD3 (polyclonal rabbit, 1:200 dilution, Agilent/Dako, catalog A0452) and anti-CD79a (clone HM57, 1:50 dilution, Agilent/Dako, catalog M7050) primary antibodies. Sections were processed using a polymer-based detection system (EnVision+ Dual Link, Dako) with 3,3′-diaminobenzidine (DAB) as chromogen. Cases were classified as B-cell when >50% of neoplastic cells expressed CD79a with absent CD3 expression, and as T-cell when >50% expressed CD3 with absent CD79a.

### 2.7. Clonality Assessment by PARR

Clonality was confirmed in all G2 cases by PARR performed on DNA extracted from lymph node aspirated samples. PCR amplification targeted the immunoglobulin heavy chain (IgH) and T-cell receptor gamma (TRG) gene rearrangements using previously described primer sets [17]. Clonal rearrangements were identified by the presence of dominant PCR products, confirming neoplastic lymphoid proliferation.

### 2.8. Lymph Node Assessment

The largest diameter of affected peripheral lymph nodes was measured using a caliper at T0 and again at T1. Measurements were used to categorize chemotherapy response based on dimensional criteria.

### 2.9. Treatment Protocol and Response Evaluation

All dogs in G2 received a standard 19-week CHOP-based chemotherapy protocol, consisting of cyclophosphamide, doxorubicin, vincristine, and prednisolone. Dose modifications were implemented according to VCOG guidelines for hematologic or gastrointestinal toxicities [18]. In T-cell lymphoma cases, CHOP-based chemotherapy was initiated due to delayed immunophenotyping results, reflecting real-world clinical practice.

Chemotherapy response was evaluated at T1 according to adapted Veterinary Cooperative Oncology Group (VCOG) response criteria.

Responses were classified as: complete response (CR) when there was complete disappearance of measurable disease or normalization of lymph node size (≤5 mm); partial response (PR) with ≥30% reduction in lymph node diameter; progressive disease (PD) when there was ≥20% increase in lymph node diameter or appearance of new lesions; or stable disease (SD): when changes did not meet criteria for CR, PR, or PD.

### 2.10. Statistical Analysis

Sample size calculation was performed a priori using G*Power 3.1 software (University of Düsseldorf, Düsseldorf, Germany). To detect a large effect size (Cohen’s d = 0.8) between groups with 80% power and α = 0.05, a minimum of 7 dogs per group was required for independent samples comparisons. For paired comparisons (T0 vs. T1), a minimum of 15 dogs was needed.

Sample size for G1 (healthy controls) was determined based on resource availability and feasibility of recruiting healthy client-owned dogs willing to undergo venipuncture. Given that the observed difference in LDH between groups was extremely large (median 143 vs. 545.5 U/L; Cohen’s d = 1.89), our sample size was adequate for the primary comparison. Although our G2 sample (n = 20) exceeded the minimum required, the study may have been underpowered to detect associations with less frequent clinical variables.

All statistical analyses were performed using R software 4.3.1 (R Core Team^®^, 2023). Due to sample size limitations and non-normal data distribution, non-parametric methods were applied.

The Kruskal–Wallis test compared serum LDH concentrations between G1 and G2.

The Wilcoxon signed-rank test evaluated changes in LDH levels within G2 between T0 and T1.

Pearson’s correlation was used to examine associations between LDH concentrations and continuous variables such as ionized calcium levels, lymph node size, and PCV.

Variables showing significant associations were included as fixed effects in mixed-effects regression models, with animal ID as a random effect to account for repeated measures.

Statistical significance was set at *p* ≤ 0.05 for all comparisons.

## 3. Results

### 3.1. Study Population

The lymphoma group (G2) included 20 dogs diagnosed with high-grade ML. The group had a median age of 8.0 years (range: 3–12), median body weight of 15.5 kg (range: 4.0–48.0), and comprised 4 males (20%) and 16 females (80%), with 14/16 females (87.5%) spayed. Clinical staging distribution was stage III (n = 8, 40%), stage IV (n = 7, 35%), and stage V (n = 5, 25%). Substage distribution showed 12 dogs (60%) as substage A and 8 dogs (40%) as substage B.

At diagnosis, hematological abnormalities included: anemia (13/20, 65%), thrombocytopenia (5/20, 25%), and leukocytosis (6/20, 30%). Biochemical abnormalities included hypoalbuminemia (7/20, 35%) and elevated ALT (9/20, 45%).

Immunophenotyping by immunohistochemistry (IHC) revealed 18/20 (90%) dogs with B-cell lymphoma (CD79a+/CD3-) and 2/20 (10%) with T-cell lymphoma (CD3+/CD79a-). Among B-cell cases, histologic classification identified 16/18 (88.9%) as diffuse large B-cell lymphoma (DLBCL) and 2/18 (11.1%) as lymphoblastic lymphoma. The two T-cell cases were classified as peripheral T-cell lymphoma-not otherwise specified (PTCL-NOS).

### 3.2. Clinical Staging and Substaging

Clinical staging according to WHO criteria revealed the following distribution: Stage III or probable Stage III (single or multiple lymph nodes in ≥2 regional areas, without confirmed bone marrow evaluation, n = 8, 40%), Stage IV or probable Stage IV (liver and/or spleen involvement, without confirmed bone marrow evaluation, n = 7, 35%).

Bone marrow infiltration was defined cytologically as ≥20% lymphoblasts among all nucleated cells on bone marrow aspirate evaluation, consistent with established criteria for cytologic assessment of bone marrow involvement and WHO Stage V disease in canine lymphoma [19]. Of the eight dogs evaluated, five (62.5%) met this threshold and were therefore classified as Stage V (5/20; 25% of the total study population). No cases of Stage I or II disease were identified in this cohort.

Bone marrow aspiration was conducted in 8/20 dogs (40%) based on the presence of thrombocytopenia (n = 5), severe anemia or circulating atypical lymphocytes (n = 3). Among dogs not undergoing bone marrow examination (n = 12), clinical stages III and IV should be considered putative classifications, as occult bone marrow involvement cannot be definitively excluded.

Substage classification showed 12 dogs (60%) as substage A (no systemic clinical signs) and 8 dogs (40%) as substage B (presence of systemic clinical signs including lethargy, anorexia, weight loss, or fever).

No significant associations were observed between clinical stage (III vs. IV vs. V; *p* = 0.421, Kruskal–Wallis test) or substage (A vs. B; *p* = 0.618, Mann–Whitney U test) and baseline LDH levels.

### 3.3. Comparison of LDH Levels Between Groups

**Figure 1:**
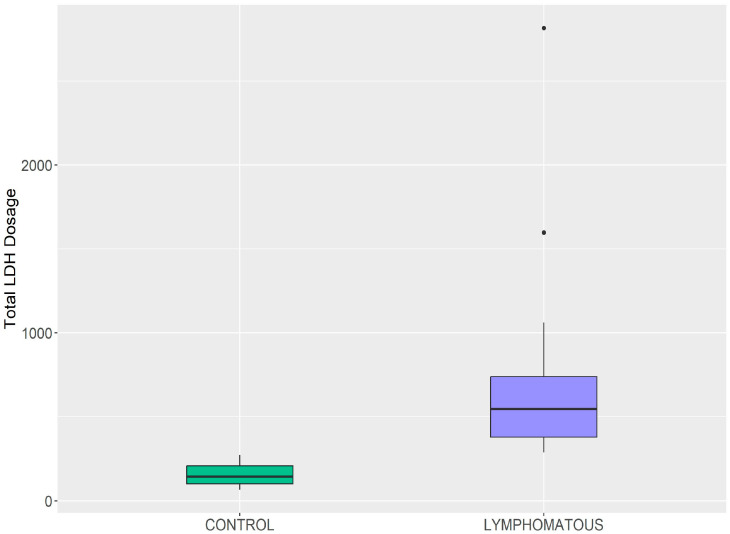
Comparison of serum lactate dehydrogenase (LDH) concentrations between healthy dogs (G1, n = 7) and dogs with high-grade multicentric lymphoma (G2, n = 20) at baseline. Box plots show median (horizontal line), interquartile range (box), and range (whiskers). *p* < 0.001, Kruskal–Wallis test. The black dots show individual data points that fall outside the interquartile range.

Dogs with high-grade ML (G2) exhibited significantly higher serum LDH concentrations at baseline (median 545.5 U/L; interquartile range [IQR]: 360.8–721.6 U/L; range: 288.2–2816 U/L) compared to healthy controls (median 143.0 U/L; IQR: 107.0–206.0 U/L; range: 66–272 U/L; *p* < 0.001, Kruskal–Wallis test) (Figure 1).

### 3.4. LDH Levels and Clinical Variables

Thrombocytopenic dogs demonstrated significantly higher median LDH levels) compared to non-thrombocytopenic counterparts at T0 (746.0 vs. 485,0 U/L; range: 612–921 U/L *p* = 0.006). Additionally, dogs with thrombocytopenia exhibited a significantly greater reduction in LDH levels between T0 and T1 (median reduction: −1011.7 U/L; range: −159 to −2064 U/L; *p* = 0.004, Kruskal–Wallis test) (Table 1).

Clinical stage, substage, reproductive status, gender, cachexia, and anemia did not show statistically significant associations with LDH concentrations or changes post-chemotherapy (Table 1).

### 3.5. Characterization of Thrombocytopenic Dogs

Among the five thrombocytopenic dogs, clinical staging distribution was as follows: Stage III (n = 1, 20%), Stage IV (n = 2, 40%), and Stage V (n = 2, 40%). Four dogs (80%) were classified as substage B. The median platelet count in this subgroup was 142,000/μL (range: 98,000–168,000/μL). All thrombocytopenic dogs tested negative for vector-borne diseases. This subgroup demonstrated significantly higher baseline LDH levels (median 746.0 U/L; range: 612–921 U/L) and exhibited more pronounced treatment-related LDH reductions (median −68.3%; range: −52.1% to −81.4%; *p* = 0.004), suggesting that thrombocytopenia might be correlated with greater metabolic activity and tumor burden.

### 3.6. Treatment Response and LDH Dynamics

At T1 (week 6), treatment response was assessed in 15/20 dogs. Five dogs were not evaluated at this time point: three exhibited progressive disease with inadequate response to CHOP-based chemotherapy and required early transition to rescue protocols before completing the 6-week evaluation period, and two owners elected to discontinue treatment due to financial or personal constraints. Among the 15 dogs evaluated, treatment response distribution was: CR 9/15 (60%); PR 3/15 (20%); and PD 3/15 (20%).

Among dogs achieving CR, LDH normalized in 7/9 (77.8%), whereas 2/9 (22.2%) retained mild residual elevations (median 312 U/L; range: 312–318 U/L). In contrast, LDH normalization was observed in only 1/6 (16.7%) dogs with PR or PD (*p* = 0.02, Fisher’s exact test). The median percentage reduction in LDH was significantly greater in dogs with CR (−63.2%; IQR: 28.5%) compared with those with PR or PD (−28.4%; IQR: 42.1%; *p* = 0.041, Mann–Whitney U test) (Table 2).

Five dogs from the original cohort (n = 20) were not evaluated at T1: three dogs exhibited progressive disease with poor response to CHOP and required early transition to rescue protocols before week 6, and two owners elected to discontinue chemotherapy.

Baseline LDH levels showed a numerical trend toward higher values in dogs that subsequently developed PD (median 748 U/L; range: 612–921 U/L) compared to those achieving CR (median 612 U/L; range: 398–846 U/L), although this difference did not reach statistical significance (*p* = 0.286, Kruskal–Wallis test), likely due to limited sample size.

### 3.7. Multivariate Analysis

Mixed-effects modeling identified a significant interaction between thrombocytopenia and timepoint (T0 vs. T1) (*p* < 0.001), confirming that thrombocytopenic dogs exhibited a steeper decline in LDH levels during chemotherapy.

No other variables demonstrated significant interactions with LDH dynamics after adjustment for repeated measures.

## 4. Discussion

This prospective study confirms that serum LDH concentrations are significantly elevated in dogs with high-grade ML compared to healthy controls. Moreover, LDH levels declined significantly following induction chemotherapy, supporting its potential utility as a dynamic biomarker for disease burden and treatment response in canine lymphoma. Clinical staging for some dogs was classified as probable stage III or IV due to the absence of bone marrow evaluation, reflecting real-world diagnostic constraints.

Our findings are consistent with previous reports in both human and veterinary oncology, where elevated LDH levels have been associated with tumor aggressiveness, hypoxia, and increased cellular turnover [5,6,7,8,9]. The marked difference in serum LDH concentrations between healthy dogs and those with ML reinforces the diagnostic value of LDH, particularly in the context of high-grade disease.

A limitation of measuring total LDH activity is the lack of tissue specificity, as LDH is ubiquitously expressed across multiple organs. However, the marked elevation observed in lymphoma patients, combined with significant treatment-related reductions, strongly suggests a predominant tumor-related origin. Future studies incorporating LDH isoenzyme analysis (particularly LDH-2 and LDH-3, which predominate in lymphoid tissues [6,7,8,9]) could provide greater diagnostic specificity and mechanistic insights into metabolic reprogramming in canine lymphoma.

Given the ubiquitous tissue distribution of LDH, elevated serum activity should not be interpreted as a lymphoma-specific marker, but rather as a nonspecific indicator of increased cellular turnover, tissue infiltration, and metabolic stress associated with aggressive disease.

Interestingly, factors such as reproductive status, gender, body condition score, and anemia did not significantly influence LDH levels or treatment-related changes, suggesting that LDH is more closely tied to tumor biology rather than host physiological characteristics. This finding is aligned with the concept that metabolic markers such as LDH primarily reflect intrinsic neoplastic processes, including hypoxia-induced metabolic reprogramming and immune evasion [3,4,20].

The strong link between LDH elevation and tumor hypoxia may also explain the association with thrombocytopenia observed in our cohort. Hypoxia can impair megakaryocyte differentiation and platelet production, potentially contributing to reduced platelet counts in aggressive lymphoma cases. Although we did not measure hypoxia-inducible factors (HIFs) or LDH isoenzymes (e.g., LDH-5), these markers could provide additional insights into tumor metabolism and warrant further investigation [6,9].

While serum LDH has previously been investigated in canine lymphoma, its clinical interpretation has remained largely descriptive and inconsistent across studies. The present work advances the field by reframing LDH as an early metabolic surrogate of tumor burden during induction chemotherapy rather than merely a static or diagnostic marker. By prospectively quantifying LDH dynamics within the first six weeks of CHOP-treatment, we demonstrate that the depth of LDH reduction and biochemical normalization provides information that is partially independent from morphological response assessed by lymph node size alone. This introduces the concept of biochemical response depth in canine lymphoma; a framework widely applied in human oncology but rarely explored in veterinary medicine.

Additionally, the identification of a thrombocytopenic subgroup with markedly elevated baseline LDH and pronounced treatment-related declines suggests the existence of biologically distinct metabolic phenotypes, generating testable hypotheses regarding tumor hypoxia, proliferation, and marrow involvement. Collectively, these findings position LDH as a low-cost, accessible biomarker with potential utility for early response assessment and risk stratification, and provide a rationale for future studies integrating LDH kinetics with molecular measures of minimal residual disease (MRD).

Our findings suggest several potential applications for LDH monitoring in canine lymphoma, though prospective validation is required.

First, dogs presenting with extremely elevated LDH (>800–1000 U/L) may represent candidates for clinical trials evaluating intensified or alternative first-line protocols, though our study did not prospectively test this hypothesis.

Second, failure to achieve ≥ 50% LDH reduction by week 6 could potentially indicate suboptimal response, warranting closer monitoring or treatment modification. However, the optimal LDH threshold and timepoint require validation in larger cohorts.

Third, persistent LDH elevation despite clinical complete response might reflect MRD, though this remains speculative as it was not measured in our study. Integration of LDH with validated MRD techniques (flow cytometry, circulating tumor DNA) would be necessary to test this hypothesis.

These potential applications require prospective validation before implementation in clinical practice.

Key strengths of this study include its prospective design, the inclusion of healthy controls with comparable age and breed distribution to G2 dogs, standardized sample handling to minimize preanalytical variability, and exclusion of dogs with clinically evident concurrent diseases and screening for vector-borne diseases in thrombocytopenic cases. Diagnostic accuracy was ensured through the use of PARR to confirm clonality and immunohistochemistry for immunophenotyping.

Nevertheless, several limitations must be acknowledged. Although the sample size was adequate for addressing the primary endpoints, the relatively small cohort (n = 20) may have limited the detection of associations involving less frequent variables and precluded robust subgroup analyses according to histologic subtype.

Although baseline LDH levels were numerically higher in dogs requiring rescue therapy, our study was underpowered to definitively establish LDH as a predictor of treatment failure. Larger prospective studies are needed to determine whether extremely elevated baseline LDH (>800–1000 U/L) identifies dogs unlikely to respond to CHOP-based chemotherapy who might benefit from alternative first-line regimens.

Follow-up was restricted to the induction phase, with LDH measurements obtained only at baseline and week 6. This timeframe was selected because most treatment responses and disease recurrences in canine lymphoma occur during or immediately after induction chemotherapy. Additionally, practical constraints including client compliance, cost considerations, and the primary study objective of evaluating early treatment response limited our ability to obtain serial measurements throughout the entire 19-week protocol. Notably, three dogs required early transition to rescue protocols before week 6 due to progressive disease, and two owners discontinued treatment, resulting in an attrition rate of 25% (5/20 dogs) before the first response evaluation.

This attrition highlights the aggressive nature of canine high-grade ML and represents a limitation, as these dogs with the most refractory disease could not be included in the dynamic LDH analysis. Extended monitoring during maintenance therapy, relapse, and survival would be necessary to validate LDH as a predictor of long-term outcomes. Furthermore, the assessment of total LDH does not allow differentiation of tissue origin, as LDH comprises five isoenzymes with distinct tissue distributions. Isoenzyme profiling could improve diagnostic specificity by distinguishing tumor-related LDH elevations from those secondary to hepatopathy, hemolysis, or muscle injury; however, total LDH measurement remains the most accessible approach in routine veterinary practice, and the exclusion of hemolyzed samples and dogs with clinically relevant hepatopathy partially mitigates this concern.

Another limitation of this study is that only LDH levels and lymph node dimensions were assessed at T1, without repeating the complete hematological and biochemical panel performed at baseline. Serial measurement of all clinical variables would have provided more comprehensive data for correlation analysis and potentially strengthened the interpretation of LDH dynamics. Future studies should incorporate complete laboratory reassessment at multiple timepoints to better characterize the relationship between LDH changes and overall disease response.

The predominance of B-cell lymphomas in the study population (18/20, 90%) reflects the natural epidemiological distribution of canine high-grade ML and enhances the clinical applicability of our findings to the most common immunophenotype. However, the small number of T-cell cases (n = 2) precludes robust immunophenotype-specific subgroup analysis. LDH kinetics may differ between B-cell and T-cell lymphomas due to distinct metabolic characteristics and clinical behavior. Future multicenter studies with larger sample sizes should specifically evaluate LDH dynamics across immunophenotypes to determine whether the patterns observed in our predominantly B-cell cohort extend to T-cell lymphomas.

Two dogs in our cohort had T-cell immunophenotype (PTTCL-NOS). While CHOP-based protocols might not be optimal for T-cell lymphoma, these cases were treated with CHOP due to delayed immunophenotyping results, reflecting real-world clinical practice. Only one T-cell case was evaluated at T1 (LDH reduction from 589 to 398 U/L with PR), limiting conclusions regarding LDH kinetics in T-cell lymphoma. Future studies should specifically evaluate LDH dynamics in T-cell lymphomas treated with T-cell-optimized protocols (e.g., LOPP, lomustine-based regimens).

A limitation of this study is the lack of immunophenotypic characterization of circulating immune cell populations. Myeloid-derived suppressor cells (MDSCs), in particular, have been implicated in metabolic reprogramming, immunosuppression, and increased glycolytic activity in aggressive lymphomas and could represent a potential cellular source of elevated LDH. The integration of LDH kinetics with flow cytometric or molecular assessment of myeloid populations, including MDSCs, should be explored in future prospective studies to better elucidate the immunometabolic mechanisms underlying LDH elevation in canine lymphoma.

Finally, the results may not be fully generalizable to populations with different breed distributions, treatment protocols, or endemic infectious diseases. Multicenter studies with larger sample sizes, longer follow-up periods, incorporation of LDH isoenzyme analysis, and inclusion of T-cell lymphomas are warranted to more definitively establish the prognostic and predictive value of LDH in canine lymphoma.

## 5. Conclusions

This prospective study confirms that serum LDH is significantly elevated in dogs with high-grade ML compared to healthy controls and declines following effective CHOP-based chemotherapy for six weeks.

Thrombocytopenic dogs exhibited higher baseline LDH levels and more pronounced treatment-related reductions, suggesting that thrombocytopenia may identify a subset with greater metabolic activity and tumor burden. Dogs achieving CR were significantly more likely to normalize LDH levels compared to those with PR or PD.

These findings support the utility of LDH as an accessible, cost-effective biomarker for assessing treatment response in canine ML during the early induction phase. However, validation in larger cohorts with extended follow-up is needed to establish LDH’s prognostic value for predicting relapse and survival outcomes. Integration of LDH with molecular biomarkers such as circulating tumor DNA may further enhance risk stratification and guide personalized treatment strategies.

## Figures and Tables

**Table 1:** Association between baseline serum LDH levels and clinical variables in dogs with high-grade lymphoma.

Variable	Category	n	LDH at T0, Median (IQR), U/L	*p*-Value
Thrombocytopenia	Yes	5	746.0 (612.0–921.0)	0.006 **
	No	15	485.0 (352.0–598.0)	
Clinical stage	Probable Stage III	8	512.0 (398.0–624.0)	0.421
	Probable Stage IV	7	587.0 (445.0–712.0)	
	Confirmed Stage V	5	678.0 (589.0–798.0)	
Substage	A	12	523.0 (412.0–645.0)	0.618
	B	8	598.0 (478.0–734.0)	
Anemia	Yes	13	558.0 (432.0–698.0)	0.712
	No	7	512.0 (389.0–621.0)	
Sex	Male	4	534.5 (412.0–678.0)	0.892
	Female	16	551.0 (398.0–723.0)	
Body condition	Underweight (BCS < 4)	11	578.0 (445.0–734.0)	0.456
	Normal/Overweight	9	512.0 (378.0–645.0)	

IQR, interquartile range; LDH, lactate dehydrogenase; BCS, body condition score. ** *p* < 0.01, Kruskal–Wallis test. Reference interval for LDH: 10–280 U/L.

**Table 2:** Serum LDH dynamics stratified by treatment response at week 6 of chemotherapy (n = 15).

Treatment Response	LDH T0 (U/L)	LDH T1 (U/L)	Δ LDH (%)	LDH Normalized, n (%)
Complete response	612 (398–846)	246 (178–318)	−63.2 (−78.0 to −48.0)	7/9 (77.8%)
Partial response	573 (412–689)	412 (324–486)	−28.1 (−42.0 to −18.0)	1/3 (33.3%)
Progressive disease	748 (612–921)	586 (448–712)	−21.7 (−35.0 to −8.0)	0/3 (0%)
*p*-value	—	0.286	0.012	0.020

Values are presented as median (interquartile range) unless otherwise stated. Δ LDH, percentage change from baseline; LDH normalization defined as ≤280 U/L at T1; T0, diagnosis/baseline; T1, week 6 of chemotherapy.

## Data Availability

The data presented in this study are available on request from the corresponding author due to ethical restrictions (patient confidentiality).

## References

[1] Wu Y., Lu C., Pan N., Zhang M., An Y., Xu M., Zhang L., Guo Y., Tan L. (2021). Serum Lactate Dehydrogenase Activities as Systems Biomarkers for 48 Types of Human Diseases. Sci. Rep..

[2] Klein R., Nagy O., Tóthová C., Chovanová F. (2020). Clinical and Diagnostic Significance of Lactate Dehydrogenase and Its Isoenzymes in Animals. Vet. Med. Int..

[3] Liberti M.V., Locasale J.W. (2016). The Warburg Effect: How Does It Benefit Cancer Cells?. Trends Biochem. Sci..

[4] Barba I., Carrillo-Bosch L., Seoane J. (2024). Targeting the Warburg Effect in Cancer: Where Do We Stand?. Int. J. Mol. Sci..

[5] Claps G., Faouzi S., Quidville V., Chehade F., Shen S., Vagner S., Robert C. (2022). The Multiple Roles of LDH in Cancer. Nat. Rev. Clin. Oncol..

[6] Dumontet C., Drai J., Bienvenu J., Berard N., Thieblemont C., Bouafia F., Bayle F., Moullet I., Salles G., Coiffier B. (1999). Profiles and Prognostic Values of LDH Isoenzymes in Patients with Non-Hodgkin’s Lymphoma. Leukemia.

[7] Wulaningsih W., Holmberg L., Garmo H., Malmstrom H., Lambe M., Hammar N., Walldius G., Jungner I., Ng T., Van Hemelrijck M. (2015). Serum Lactate Dehydrogenase and Survival Following Cancer Diagnosis. Br. J. Cancer.

[8] Kottmann R.M., Kulkarni A.A., Smolnycki K.A., Lyda E., Dahanayake T., Salibi R., Honnons S., Jones C., Isern N.G., Hu J.Z. (2012). Lactic Acid Is Elevated in Idiopathic Pulmonary Fibrosis and Induces Myofibroblast Differentiation via PH-Dependent Activation of Transforming Growth Factor-β. Am. J. Respir. Crit. Care Med..

[9] Zanatta R., Abate O., D’angelo A., Miniscalco B., Mannelli A. (2003). Diagnostic and Prognostic Value of Serum Lactate Dehydrogenase (LDH) and LDH Isoenzymes in Canine Lymphoma. Vet. Res. Commun..

[10] Marconato L., Crispino G., Finotello R., Mazzotti S., Salerni F., Zini E. (2009). Serum Lactate Dehydrogenase Activity in Canine Malignancies. Vet. Comp. Oncol..

[11] Marconato L., Crispino G., Finotello R., Mazzotti S., Zini E. (2010). Clinical Relevance of Serial determinations of Lactate Dehydrogenase Activity used to Predict Recurrence in Dogs with Lymphoma. J. Am. Vet. Med. Assoc..

[12] Zandvliet M. (2016). Canine Lymphoma: A Review. Vet. Q..

[13] Lucroy M.D., Christopher M.M., Kraegel S.A., Simonson E.R., Madewell B.R. (1998). Anaemia Associated with Canine Lymphoma. Comp. Haematol. Int..

[14] Clarke H., Pallister C.J. (2005). The Impact of Anaemia on Outcome in Cancer. Clin. Lab. Haematol..

[15] Coady M., Fletcher D.J., Goggs R. (2019). Severity of Ionized Hypercalcemia and Hypocalcemia Is Associated with Etiology in Dogs and Cats. Front Vet. Sci..

[16] Owen L.N., Owen L.N. (1980). TNM Classification of Tumours in Domestic Animals.

[17] Burnett R.C., Vernau W., Modiano J.F., Olver C.S., Moore P.F., Avery A.C. (2003). Diagnosis of Canine Lymphoid Neoplasia Using Clonal Rearrangements of Antigen Receptor Genes. Vet. Pathol..

[18] Vail D.M., Michels G.M., Khanna C., Selting K.A., London C.A. (2010). VCOG Response Evaluation Criteria for Peripheral Nodal Lymphoma in Dogs (v1.0)—A Veterinary Cooperative Oncology Group (VCOG) Consensus Document. Vet. Comp. Oncol..

[19] Messick J.B. (2023). A Primer for the Evaluation of Bone Marrow. Vet. Clin. N. Am.—Small Anim. Pract..

[20] Hanahan D. (2022). Hallmarks of Cancer: New Dimensions. Cancer Discov..

